# Evaluation of a Mindfulness-Based Mobile App Aimed at Promoting Awareness of Weight-Related Behaviors in Adolescents: A Pilot Study

**DOI:** 10.2196/resprot.6695

**Published:** 2017-04-26

**Authors:** Tami Turner, Melanie Hingle

**Affiliations:** ^1^ Department of Nutritional Sciences The University of Arizona Tucson, AZ United States

**Keywords:** mindfulness, adolescent, mHealth, diet, physical activity, app

## Abstract

**Background:**

Mindfulness-based interventions are reported to be highly acceptable and have positive effects on youth, yet most are clinic- or school-based aimed at emotional regulation or academic performance. To provide flexible program delivery, we developed and tested a standalone mindfulness-based app aimed at improving weight-related behaviors (eg, diet, physical activity, sleep) in adolescents.

**Objective:**

Our objective was to assess the feasibility, acceptability, and utility of a mindfulness-based mobile app.

**Methods:**

In a single-arm pilot study, 15 adolescents (14-18 years) were prompted to access the app once a day, every day for 6 weeks. Outcomes were measured by in-app and poststudy surveys, and descriptive statistical analyses were performed. Time within a mindfulness state was self-reported during weekly timed practices.

**Results:**

The app was rated highly for content and encouraging the practice of activities to promote mindfulness states. Teens reported increased awareness of eating behaviors and high adherence, particularly during physically active practices. Average self-reported time spent in a mindfulness state increased 2.5 times by week 6 (78 [SD 17] seconds) compared to week 1 (31 [SD 21] seconds).

**Conclusions:**

The high acceptability and utility ratings of the app, increases in reported time in mindfulness states, and high frequency of participation, including mindful eating and physical activity, suggest the mindfulness-based mobile app has the potential to improve awareness of weight-related behaviors.

## Introduction

The psychological state of mindfulness facilitates present moment awareness, objective self-observation, and attention to the environment without judgement [1-3]. Mindfulness may promote greater self-regulation, as is required for successful weight management, because intermittent self-monitoring of goals produces less effective self-regulation than does regular attention to one’s present behavior [4]. In adults, mindfulness-based interventions related to eating have increased awareness of satiety; feelings and thoughts about food and the food environment [5-8]; and reduced weight, body mass index (BMI), and caloric and fat intake [5,7]. Mindfulness-based interventions have decreased blood pressure in adolescents with increased risk of cardiovascular disease [9], improved body satisfaction and reduced disordered eating thoughts in fifth grade girls [10], improved sleep in youth with history of substance abuse [11], and increased physical activity and improved dietary habits in overweight and obese teens [12]. However, the majority of mindfulness-based interventions for youth have been school- or clinic-based programs aimed at improving emotional regulation and academic performance [13,14]. Little research has investigated the effect of mindfulness on weight-related behaviors in youth.

Acceptability of mindfulness interventions in youth is high [14,15]; however, time commitment associated with attending face-to-face programs is a frequently cited reason for nonparticipation in adolescents [16]. Research also confirms mobile health (mHealth) interventions are feasible and acceptable approaches in the prevention and treatment of pediatric obesity [17], and teens report a preference for virtual mindfulness-based health promotion programs [18]. We are unaware of mHealth programs focused on mindfulness to increase awareness of weight-related behaviors in adolescents. The objective of this study was to evaluate the acceptability, feasibility, and utility of a mindfulness-based intervention delivered via mobile app in teens aged 14 to 18 years.

## Methods

### App Development

A series of videos was created using animation software (GoAnimate.com) and integrated into an established mobile- and cloud-based app platform developed by our industry partners at Vignet, Inc, thereby creating the b@Ease Mindfulness App. The mobile app and its cloud server are secured and compliant with the Health Insurance Portability and Accountability Act and the Health Information Technology for Economic and Clinical Health Act to protect participant privacy and confidentiality.

Elements of face-to-face mindfulness-based programs were integrated into the videos, including self-observation skill-building, increasing awareness of hunger/satiety cues and the sensory aspects of eating, and purposefully paying attention to physical movement of the body. Regardless of the method used to focus attention (eg, breath), intervention content emphasized achieving and remaining in a mindful state. Videos used fictional storytelling and analogy to communicate, presented techniques to evoke a state of mindfulness, encouraged participation during guided practices, and challenged teens to integrate mindfulness into life as independent practice. Videos range from 2 to 15 minutes (mean 7.5 [SD 2.8] minutes).

Guided practices occurred on 37 days (88% of videos), with mindful eating and physical movement practices presented on 18 days (43% of videos). Textbox 1 summarizes video topics, techniques, and storytelling examples.

**Content of videos within the b@Ease Mindfulness App.**

Topics:

- General mindfulness
- Sleep
- Mindful eating
- Physical activity
- Stress
- Social/relationships

Techniques to initiate mindfulness states:

- Meditation
- Guided imagery
- Mindful eating
- Sensory (eg, auditory, visual)
- Body scans (eg, hunger, emotions)
- Progressive muscle relaxation
- Seated and standing stretching and movement
- Yoga and stationary standing postures (eg, balancing)
- Mindful walking and martial arts forms

Story examples:

- Hippo with insomnia learns breath meditation
- Talking body parts argue over who’s really hungry; mindful eating instruction follows
- Alien dies from mindless eating accident; how to prevent being a victim with mindful eating
- Boyfriend assumes mate is cheating but learns thoughts are not necessarily true; a sensory guided practice follows
- Frankenstein’s monster complains about achy body and learns mindful stretching
- Boy catastrophizing over a future public speaking event learns mindful walking

### Recruitment and Enrollment

Adolescents were recruited from Tucson, Arizona, via flyers posted at community organizations and online (eg, Facebook). Eligibility criteria included teens aged 14 to 18 years willing to participate and use personal mobile devices to use the study app and able to read and speak fluent English. Exclusion criteria included psychological pathologies (depression, anxiety disorders, posttraumatic stress disorder, schizophrenia, bipolar disorder), conditions that affect attention or mood (attention deficit hyperactivity disorder, medications), trauma, epilepsy, and disordered eating behaviors. The Patient Health Questionnaire (PHQ-4) [19] was used to screen for anxiety and depression, the Eating Attitudes Test (EAT-26) [20] for eating disorders, and participants were asked if they had experienced or were diagnosed with or seen by a professional for the remaining conditions. While body weight status (eg, obesity) was not included as an eligibility criterion, it was collected to understand more about the teens who chose to participate. Interested teens provided parental-permitted assent or consent and were screened using an online, encrypted, password-protected survey distributed via Qualtrics survey software sent as a link to the participant’s email. Participants received up to $50 for completing study procedures. The Institutional Review Board of The University of Arizona, Tucson, Arizona, approved the study.

### Intervention

At baseline, participants self-reported height, weight, gender, race, and ethnicity. Participants were asked if they had previously heard of mindfulness and whether they had participated in yoga, meditation, guided imagery, body scans, internal martial arts (eg, Ba Gua, Tai Chi), or other mind-body techniques. Participants downloaded the app onto their mobile devices, registered the app, and were asked to access the app every day for 6 weeks. To prevent the study from extending beyond 6 weeks, participants were only able to view one video per day and unable to skip or return to previous videos and the corresponding in-app surveys. Daily prompts encouraged video viewing each morning (streamed from YouTube), and a postvideo in-app survey requested self-reported adherence to video viewing and acceptability (based on a 5-point Likert scale). If the survey remained incomplete by 8 PM, an additional notification was sent. In-app surveys also assessed whether participants engaged in independent practices at least weekly on 8 separate occasions. If the participant indicated they tried the independent practice, a follow-up question asked if it was helpful or enjoyable. The poststudy survey included questions on facilitators and barriers to performing mindful eating and physical activity, likelihood of adopting mindfulness techniques, perceived utility of the content within the app, and app functionality ratings.

Once a week, a scheduled video led participants through a guided timed practice to estimate how long they were able to remain in a mindfulness state. Following the video, participants reported the number of times they were *not* in a mindful state during the practice (ie, when they caught themselves thinking of something else and had to start over). Durations were computed as the sum of the intervals of time within a mindfulness state during the practice (eg, if during a 3-minute timed practice they started over twice, 3 periods resulted, and 1-minute average time was computed). Timed sessions progressively increased from 3 to 15 minutes over the study duration.

Acceptability and utility were defined as interest and enjoyment, perceived usefulness, perceived influence on behavior and/or affect, likelihood of adoption of mindfulness techniques, and app functionality. Feasibility was defined as the capacity of the app to engage participants measured by adherence to recommended app use and participant-reported facilitators and barriers to mindful eating and physical activity practices. Data collected within the app included video ratings (based on a 5-point Likert scale) and frequency of adherence to and usefulness of several guided and independent practices (eg, “Did you try it?” and “Did you like it?”) (see Multimedia Appendix 1). The poststudy survey (Multimedia Appendix 2) asked participants to assign Likert scale ratings or A to F “grades” to indicate liking of content and practices; perceived influence of the app and facilitators/barriers to mindfulness related to eating, physical activity, and sleep-related activities; likelihood of adopting mindfulness as a practice; ease of use; and technical ratings of the app. Free text comments for suggestions for improvement of the app were also solicited as part of the poststudy survey. Survey questions of facilitators and barriers to participating in mindful eating and physical activity were only asked if participants stated they participated in the activity at least sometimes or more frequently. Furthermore, participants were able to submit in-app comments or questions if desired (Multimedia Appendix 3).

### Statistical Analysis

Analyses were conducted using Excel 2010 (Microsoft Corp), Qualtrics survey software (Qualtrics), and SPSS version 23.0 (IBM Corp). Descriptive analyses provided central tendency and dispersion for characteristics of participants, data collected within the app, and from the poststudy survey. Fisher exact test was used to determine whether baseline participant characteristics differed between teens completing the poststudy survey and teens who did not. Statistical significance was defined at the 95% confidence level (*P*<.05, 2-tailed). Changes in self-reported time spent in a mindfulness state for participants who viewed the guided practice videos are summarized.

## Results

### Participant Characteristics

Of the 95 interested respondents, 66 provided their email address to receive a secure link to complete the screening survey. The majority of ineligible participants (43/66, 65%) indicated they may have a mental health condition or received scores on the PHQ-4 suggesting they may have depression or anxiety. A smaller number of teens (12/66, 18%) were ineligible due to scores on the EAT-26 suggesting they were affected by disordered eating behaviors.

**Figure 1:**
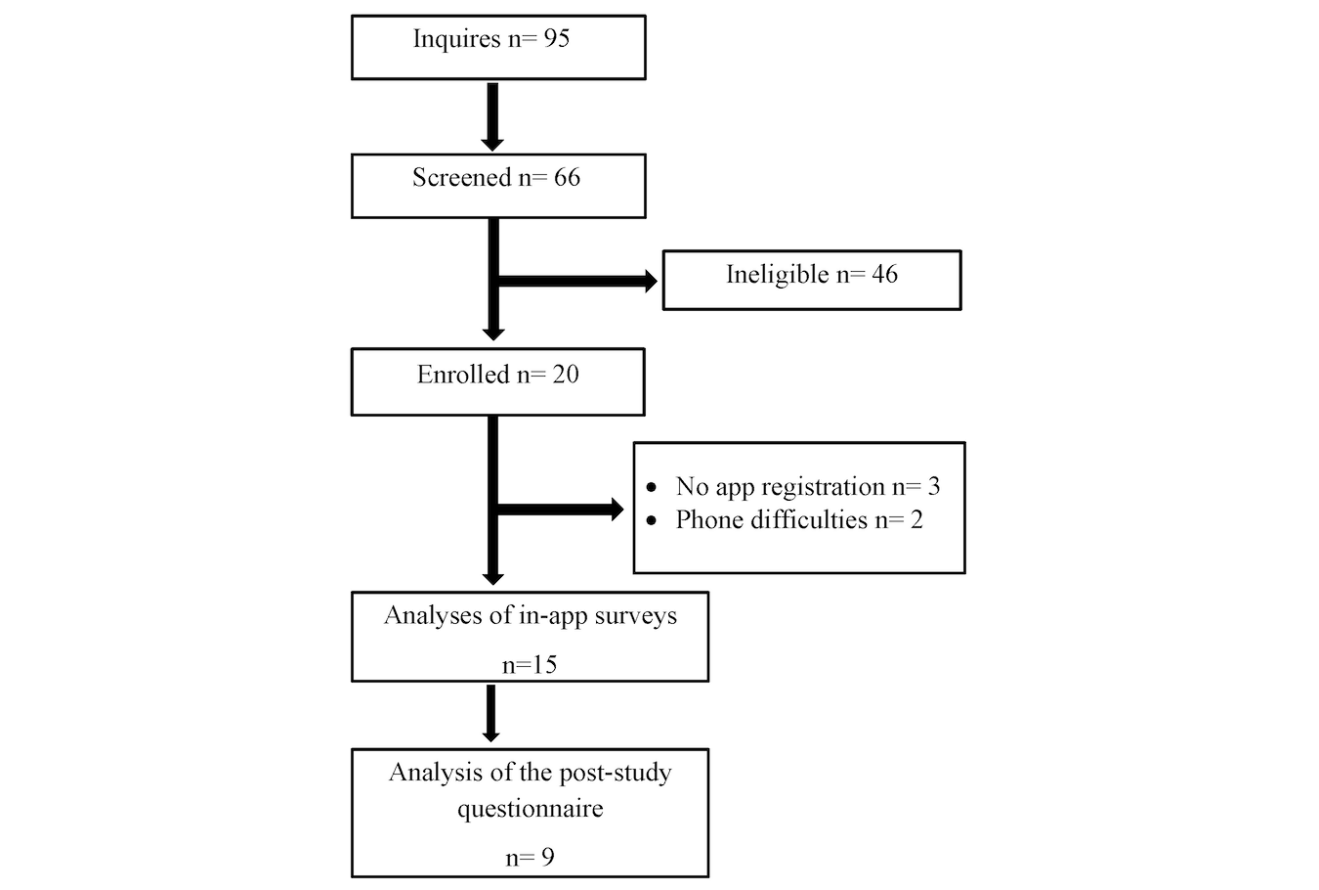
Flow of participants in the b@Ease Mindfulness App for Teens study.

Of the 66 participants screened, 20 met eligibility criteria and were enrolled (Figure 1). A total of 5 participants did not complete app registration or experienced device difficulties preventing app use. A total of 15 teens completed baseline surveys and answered surveys within the app, and 9 completed the poststudy questionnaire. Characteristics of participants who registered for the app are shown in Table 1. A high proportion of participants were Hispanic, 20% were overweight or obese, and one-third had heard of mindfulness. Of the 15 teens, 9 indicated they had performed activities such as yoga, which may or may not have been mindfully executed, indicating some may have had prior experience.

All 15 participants provided data within the app including rating videos and answering questions related to independent practices (eg, “Did you try the technique?” and “Did it help you?”), and 9 participants completed the poststudy survey and rated content, techniques, frequency of mindfulness practice, utility of the app to promote mindfulness in various ways, and likelihood of adoption. There were no differences between teens who completed the poststudy survey and teens who did not when comparing gender (*P*=.12), race (white vs mixed or nonwhite, *P*=.53), Hispanic versus non-Hispanic (*P*=.13), age group (14-16 years vs 17-18 years, *P*=.98), familiarity with mindfulness (*P*=.61), any previous practices that may have included mindfulness (*P*=.58), or weight status (normal vs overweight or obese, *P*=.77).

**Table 1:** Characteristics of the participants registered for the b@Ease Mindfulness App (n=15).

Variable		User testing
Age, years, mean (SD)		16.5 (1.4)
**Gender, n (%)**		
	Male	8 (53)
	Female	7 (47)
**Race/ethnicity^a^** **, n (%)**		
	Hispanic/Latino	9 (60)
	Not Hispanic/Latino	6 (40)
	African American	1 (7)
	Asian	2 (13)
	Native American	1 (7)
	White	14 (93)
	Unknown/refuse	2 (13)
BMI^b^ (kg/m^2^), mean (SD)		23.1 (4.3)
BMI percentile, mean (SD)		62.1 (26.1)
**Weight, n (%)**		
	Normal^c^	12 (80)
	Overweight/obese^c^	3 (20)
Heard of mindfulness, n (%)		5 (33)
Possible prior mind-body practices^d^, n (%)		9 (60)

^a^All applicable races were allowed.

^b^BMI: body mass index.

^c^Based upon BMI percentile.

^d^Choices included yoga (n=7), meditation (n=4), guided imagery (n=0), body scans (n=0), internal martials arts (eg, Chi Gong, n=1), or other mind-body techniques (n=1).

### Acceptability and Utility

**Figure 2:**
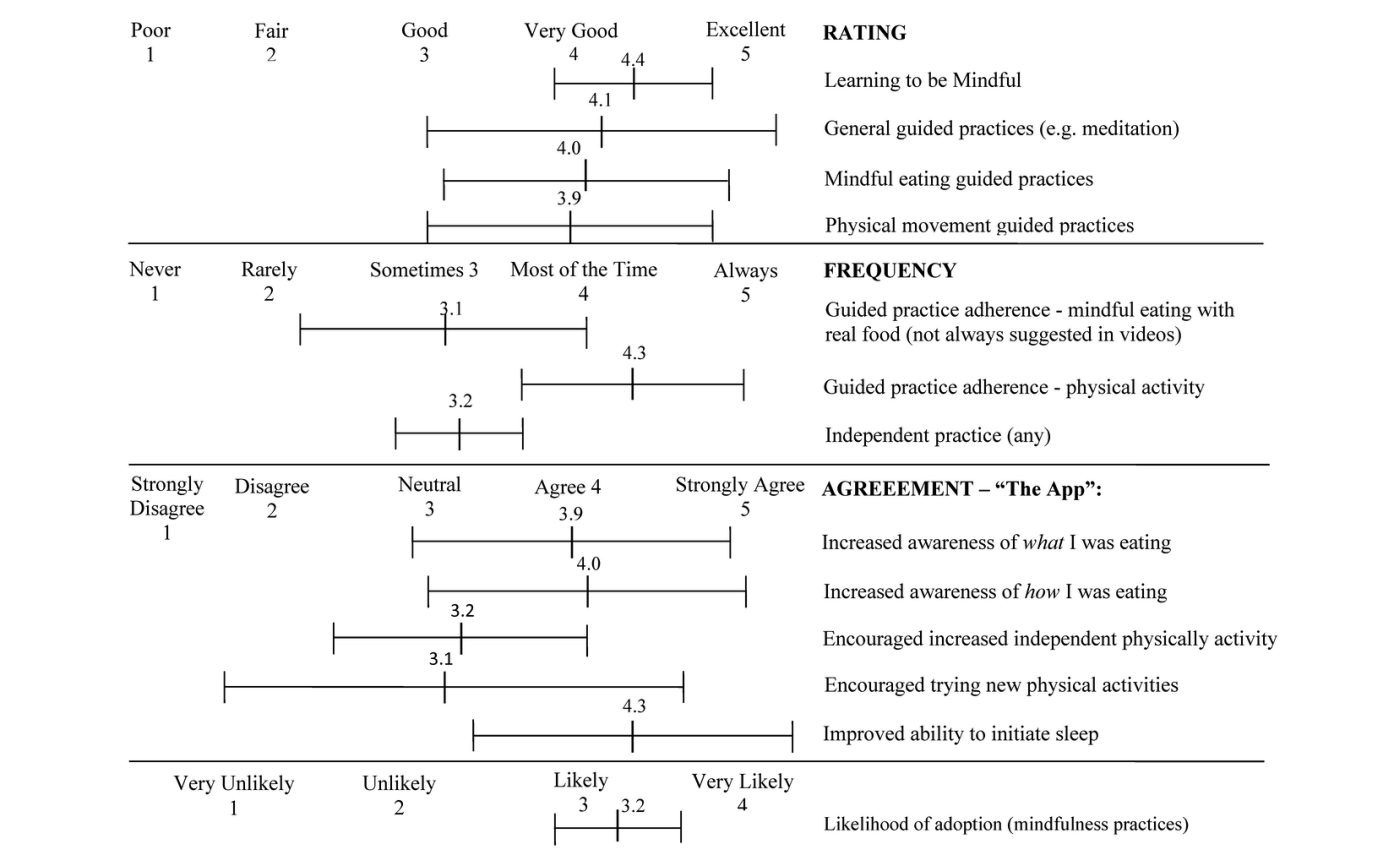
Poststudy survey results of the b@Ease Mindfulness App for Teens study (n=9, all data are mean [SD]).

Participants highly rated the videos (in-app survey mean 3.8 [SD 0.3] out of 5). The techniques, guided practices, and the utility of the app were rated highly in the poststudy survey (see Figure 2), especially videos related to physical movement, mindful eating, and sensory practices. Participants reported increased awareness of eating behaviors in poststudy surveys; comments included, “I was surprised at how much better my food tasted and how I ate less than I normally would have.” Physical activity practices were also highly rated, especially when presented as a way to appreciate and reward the body. The lowest rated videos were timed practices exceeding 8 minutes. Suggestions for improvement included shortening timed practices, allowing users to return to earlier videos, and allowing customization of app prompts to coincide with meal times. There were no reported adverse effects. Participants reported the app to be very helpful and enjoyable and reported feeling more relaxed, focused, and peaceful. Furthermore, participants stated the videos explained mindfulness as “so simple and understandable,” and users reported a high likelihood of adopting mindfulness practices. Overall, the intervention content was given an average grade of B while the functionality of the app received a B+ (scale of A-D, F).

### Feasibility

Using in-app survey responses as a proxy to measure adherence, the percentage of daily responses averaged 55% (all participants) to 73% (excluding 4 teens who stopped participating within the first few days). Thus, participants received an average of 23 (SD 17) days to 31 (SD 15) days of intervention time representing 3 to 4 hours of total viewing. All but one participant reported practicing with real food from some to every time during the guided practices focused on mindful eating; facilitators were timing of video (eg, near mealtime), desire to understand, being told to try it, and feeling appreciation for the body. Participants reported a high frequency of participation during guided physical movement-focused practices, with all but one user participating most to every time. Facilitators included being told to participate (“even though I didn't want to, I did it anyway”), increased positive outcome expectancies, and curiosity. Frequency of independent practice reported within app surveys was moderate with 50% of participants reporting sensory practices and 57% using breath meditation as a means to initiate sleep.

### Mindfulness Timed Practice

Participants increased their ability to initiate and remain in a mindful state during timed practices over the 6-week study from an average of 31 (SD 21) seconds to 78 (SD 17) seconds. Participation was highest when the duration was 10 minutes or less, and no participant attempted the 15-minute practice.

## Discussion

### Mindfulness and Health

Mindfulness promotes present moment attention to and observation of the self and environment, which in turn may facilitate self-monitoring of behavior and improve self-regulatory capacities [2,21,22]. Mindfulness-based pediatric interventions have improved dietary quality [12], increased physical activity [12], decreased stress [23], and improved sleep [11], factors associated with healthy weight management. The amount of time required to practice mindfulness to improve behaviors is unknown [13,16]. However, research suggests practicing mindfulness for 10 to 15 minutes a day may improve health outcomes [24,25].

### Principal Findings and Comparison With Prior Work

The b@Ease Mindfulness App provided 5.5 hours of mindfulness-based content and 3 hours of guided practice time over 42 days. This is similar to face-to-face mindfulness-based pediatric interventions which are often delivered less than an hour weekly for 8 to 12 weeks (8-12 days or 6-9 total hours) [13,26]. Inclusive of elements presented in face-to-face interventions, the program presents a light-hearted and entertaining way to encourage adolescents to practice achieving mindfulness states. However, unlike face-to-face programs, the b@Ease Mindfulness App provides flexible program delivery. Furthermore, the videos are easily adaptable as a program separate from the app and deliverable across a variety of platforms (eg, website). Participants in our study received 23 (SD 17) days of intervention and reported their experiences as highly acceptable and useful, and the app-based mindfulness program was feasibly delivered to this sample of teens.

### Limitations

This study demonstrates the potential for the app to increase mindfulness in teens, but our method of measuring mindfulness has not been validated and is a limitation of the study. However, participant-reported time within a mindfulness state increased over the study (even though practice times also increased), suggesting the app increased the ability of participants to initiate and sustain longer periods of mindfulness states. App survey responses were used as a proxy to measure dose. Videos were streamed from YouTube to reduce file size, and long or repeated buffering reported by a few participants increased the likelihood of the app being closed before surveys were administered. Furthermore, app-generated data indicated high receipt of commands from mobile devices, but these data do not confirm the videos were viewed. We acknowledge the small sample size of this study is a limitation; however, it provides valuable information to allow iterative development of an app involving novel techniques for teaching mindfulness.

### Conclusions

A mindfulness-based mobile app, the b@Ease Mindfulness App, was developed and tested for acceptance, utility, and feasibility in 14- to 18-year-old teens. The mobile app successfully delivered videos, which were typically viewed in less than 10 minutes every day for 6 weeks, to participants using an existing app platform. The mindfulness app was rated as an acceptable and useful program by participants, potentially providing greater reach to teens, a demographic who have expressed preference for virtual mindfulness-based health promotion programs [18]. Both comments and ratings support the app as a potential way to improve eating behaviors, encourage guided physical activity practices, facilitate sleep initiation, and improve overall well-being. Taken together, these factors may in turn improve weight-related behaviors [27,28]. Further refinement of this mobile app is warranted to confirm these posited impacts.

## Appendix

Multimedia Appendix 1 Surveys sent within the mobile app in the b@Ease Mindfulness App for Teens study.

Multimedia Appendix 2 Poststudy survey questions of the b@Ease Mindfulness App for Teens study.

Multimedia Appendix 3 Examples of comments submitted via the app by teens participating in the b@Ease Mindfulness App for Teens study.

## Abbreviations

BMI: body mass index
EAT-26: Eating Attitudes Test
mHealth: mobile health
PHQ-4: Patient Health Questionnaire
