# Supplementary material for: Evaluation of a Mindfulness-Based Mobile App Aimed at Promoting Awareness of Weight-Related Behaviors in Adolescents: A Pilot Study
Source: JMIR Res Protoc. 2017 Apr 26;6(4):e67. doi: 10.2196/resprot.6695 (PMC5425773; doi:10.2196/resprot.6695)
Supplement: Multimedia Appendix 1 [file resprot_v6i4e67_app1.pdf]

**Title**	**Surveys (in addition to daily survey*)**	**Response Options**	**Day and time sent**
Day 1 Intro and how to			
Day 2 Learn to Observe			
Day 3 Play and observe			
Day 4 Insomniac Hippo	"Did you try using the mindfulness technique to fall asleep?" Y/N; if Y "did it help you to fall asleep?"	Y/N; multiple choice "not at all, very little, some, a lot"	Day 5; 9am
Day 5 Radio Podcast			
Day 6 Body parts are taking over			
Day 7 Environment			
Day 8 Drinking	"Did you try mindful drinking during the guided practice?"	Y/N	Day 8; after video viewed
Day 9 Zombie Goes Vegan	"Did you follow along during the guided practice?"	Y/N	Day 9; after video viewed
Day 10 Alien Advice	"Did you try mindful listening on your own?"		Day 11; 9 am
Day 11 Podcast II and 3 minute challenge	"How many times were you NOT mindful during the 3 minute practice?"	text entry (numerical)	Day 11; after video viewed
Day 12 Game show and focus			
Day 13 Just Throw It In The Bag			
Day 14 Stress Leaves 5min challenge	"How many times were you NOT mindful during the 5 minute practice?"	text entry (numerical)	Day 14; after video viewed
Day 15 Alien Accident			
Day 16 Using your senses	"Did you try m eating while watching?" If yes "Did you like using mindfulness during eating?"	Y/N; "not at all, a little, some, a lot"	Day 16; after video viewed

17th Day Difficult Memories and Emotions			
Day 18 Block by block			
Day 19 Mind goggles			
Day 20 Are You Listening			
Day 21 Monster stretch			
challenge 8 minute practice	"How many times were you NOT mindful during this 8 minute practice?	text entry (numerical)	Day 22; after video viewed
Day 23 Body Hug			
Day 24 Blind Date with Your Plate			
Day 25 Superhero challenge 9 min	"How many times were you NOT mindful during this 9 minute practice?"	text entry (numerical)	Day 25; after video viewed
Day 26 Thank you and Goodnight			
Day 27 Earth and Water	"Did you follow along and do the standing practice?" If yes, "Did you like doing this practice?"	Y/N; "not at all, a little, some, a lot"	Day 27; after video viewed
Day 28 Stress and single tasking	"Did you try the standing practice challenge on your own?"	Y/N	Day 28; 9 am
Day 29 Get On Up			
Exercise your mind			
Day 31 10min Challenge	"How many times were you NOT mindful during the 10 minute practice?"	text entry (numerical)	Day 31; after video viewed
Day 32 Stress - body scan			
Day 33 Balance			
Day 34 Solo diner	"Have you tried eating mindfully?	Y/N	Day 35; 1 pm
Day 35 Walk the Dog	"Did you try the mindfulness exercise of focusing on the horizon or other objects?" If yes, "How did you feel after?"	Y/N and text entry for "how did you feel"	Day 36; 9am

Day 36 Brain Drain - challenge 12 min	"How many times were you NOT mindful during the 12 minute challenge?"	text entry (numerical)	Day 36; after video viewed
Day 37 Orale Vato and Your Muscles			
Day 38 Spinning World			
Day 39 Shift			
Day 40 Unkind Monster Mind			
Day 41 Clouds in the sky			
Last Day! 15min challenge	"How many times were you NOT mindful during the 15 minute challenge?"	text entry (numerical)	Day 42; after video viewed

*Survey on adherence and video rating for acceptability within the app sent daily shown below:

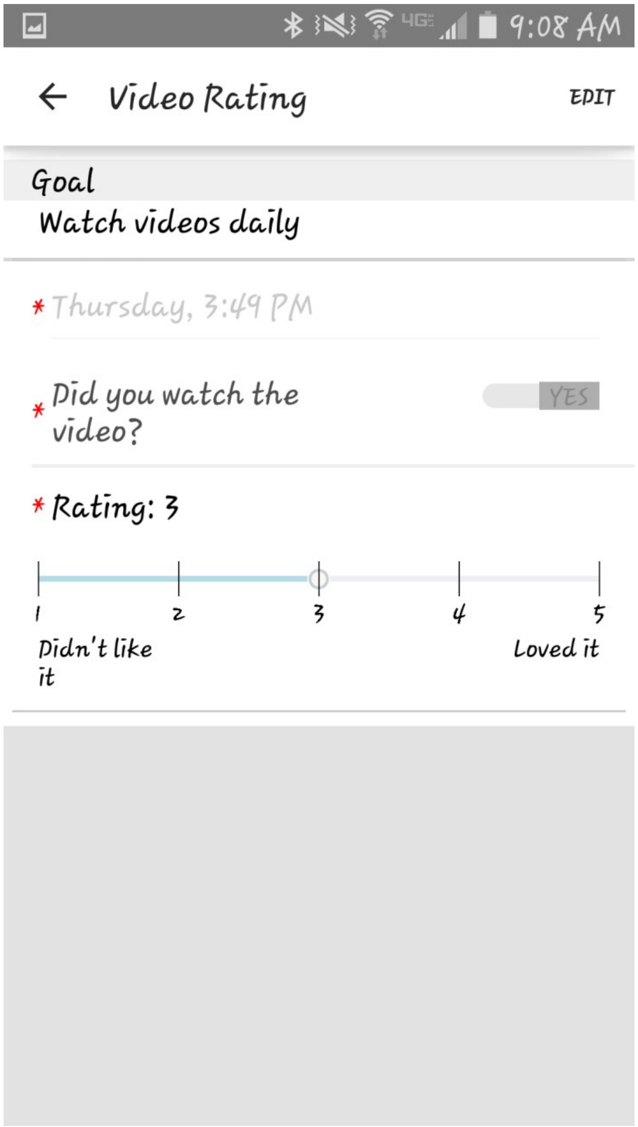
9:08 AM
Video Rating
EDIT
Goal
Watch videos daily
* Thursday, 3:49 PM
* Did you watch the video?
YES
* Rating: 3
1
2
3
4
5
Didn't like it
Loved it
