# Supplementary material for: Evaluation of a Mindfulness-Based Mobile App Aimed at Promoting Awareness of Weight-Related Behaviors in Adolescents: A Pilot Study
Source: JMIR Res Protoc. 2017 Apr 26;6(4):e67. doi: 10.2196/resprot.6695 (PMC5425773; doi:10.2196/resprot.6695)
Supplement: Multimedia Appendix 2 [file resprot_v6i4e67_app2.pdf]

Please rank the following:

	Poor	Fair	Good	Very Good	Excellent
How would you rate the guided practices that focused on doing physical movement (standing, walking, stretching)?	○	○	○	○	○
How would you rate the general mindfulness practices (like breath meditation)?	○	○	○	○	○
How would you rate the guided practices focused on eating?	○	○	○	○	○

Please rate the following the following statements by dragging the bar
This app...

	Strongly disagree 0	Disagree 1	Neither Agree nor Disagree 2	3	Agree 4	Strongly Agree 5
helped me be more physically active						
encouraged me to try new physical activities like yoga or martial arts						
helped me be more aware of WHAT I was eating						
made me be more aware of HOW I was eating						
helped me to fall asleep						

How would you rate your experience in learning how to be mindful? (Drag the bar to change the face.)

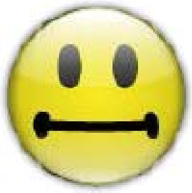

How often did you practice mindfulness during your day (without using the app)?

- Never
- Rarely
- Sometimes
- Most of the Time
- Always

How likely are you to continue practicing mindfulness?

- Very Unlikely
- Unlikely
- Likely
- Very Likely

When viewing the practices that involved mindful eating, did you try any with real food?

- Never
- Rarely
- Sometimes
- Often
- All of the Time

What were some things that motivated you to use real food during the guided mindful eating practices?

What were some things that prevented you from using real food during the mindful eating practices?

When asked to participate in the guided practices that involved physical movement such as standing in a pose, stretching, or walking, how often did follow along and do the activity as described (i.e. did you get up and actually do the movements?)

- Never
- Rarely
- Sometimes
- Often
- All of the Time

What were some things that motivated you to get up and do physical movement (stretching, standing, walking) during the guided practices?

What were some things that prevented you from doing physical movement (stretching, walking, standing) during the guided practices?

Please rate the overall CONTENT of the mindfulness-based program. In other words, overall how would you rate the videos, guided practices, and interaction you experienced (like giving your opinion and answering questions).

(Drag the bar to grade.)

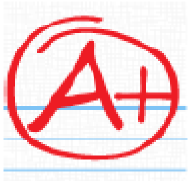

Please rate the app for how easy it was for you to use.

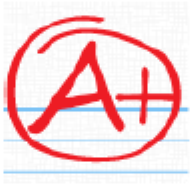

Please rate the following by dragging the bar to your choice:

Strongly Disagree | Disagree | Neither Agree nor Disagree | Agree | Strongly Agree

0 1 2 3 4 5

The app had a lot of technical issues

What would make this app better?

Do you wish that the program included something more that could have helped you stay on track?

- ○ Yes
- ○ No

What would have helped you to stay on track?
