# Supplementary material for: Evaluation of a Mindfulness-Based Mobile App Aimed at Promoting Awareness of Weight-Related Behaviors in Adolescents: A Pilot Study
Source: JMIR Res Protoc. 2017 Apr 26;6(4):e67. doi: 10.2196/resprot.6695 (PMC5425773; doi:10.2196/resprot.6695)
Supplement: Multimedia Appendix 3 [file resprot_v6i4e67_app3.pdf]

Participant 2:

- While being mindful, I was incredibly relaxed, and I almost fell asleep! The only thing that I think could be improved is the sound signaling the end. I almost didn't hear it because it was so quiet.
- I loved this video because of how lively it was! And I accept the mindful eating challenge!
- Though I haven't had breakfast yet, I tried eating my dinner last night mindfully. When I did, I was surprised at how much better my food tasted, and how I ate less than I normally would have.
- This video was very amusing, with the Orale Vato segment!

Participant 5:

- I'm really excited to learn about mindfulness and how it can help me. I tend to over analyze sometimes, so I fear it will be a bit difficult for me
- I'm starting to understand what mindfulness is, but I'm having a bit of a hard time quieting down my brain do it correctly
- This concentration method was very helpful! I'll try this during school!

Participant 6:

- I enjoy practicing mindfulness, especially when I'm just going through life and not really stopping to smell the roses.
- Mindfulness helps me to take my time and actually be in the present moment!
- As always, I liked the animations! They helped perk up the video!
- I loved the technique using the warm water! Even though I was kind of cold, I could almost feel the water inside me!

Participant 11:

- makes me feel relaxed. I like!
- I liked this video because it helped me focus on what was around me.
- I fully noticed the colors in my room, and I was surprised that I hadn't noticed them that way before!
- it was good but repetitive. it felt more like 3 good videos smashed together

Participant 12:

- Sorry I haven't been able to complete some of the surveys because it thinks that I haven't watched or rated the video. I've tried closing the app and coming back to it after a few minutes, but it still hasn't reset. thank you
- I liked the animations!!
- this is really helpful especially for someone like me
- the videos about food really don't pertain to me
- It gave me a way to practice mindfulness and it actually worked for a bit, I was very surprised, and since it gave me a way to regard the thoughts.
- I am surprisingly calm
